# Is being a “small fish in a big pond” bad for students´ psychosomatic health? A multilevel study on the role of class-level school performance

**DOI:** 10.1186/s12889-018-5977-5

**Published:** 2018-09-06

**Authors:** Katharina Rathmann, Ludwig Bilz, Klaus Hurrelmann, Wieland Kiess, Matthias Richter

**Affiliations:** 1 Department of Nursing and Health, Fulda University of Applied Sciences, Leipziger Straße 123, 36037 Fulda, Germany; 2 Department for Sociology of Rehabilitation, Faculty of Rehabilitation Sciences, Technical University Dortmund, Emil-Figge-Str. 50, 44227 Dortmund, Germany; 3 Department of Health Sciences, Faculty for Health, Social Work, and Music, Brandenburg University of Technology Cottbus-Senftenberg, Universitätsplatz 1, 01968 Senftenberg, Germany; 4 Hertie School of Governance, Friedrichstr. 180, 10117 Berlin, Germany; 5 Department of Women and Child Health, Hospital for Children and Adolescents and Centre for Paediatric Research (CPL), University of Leipzig, Liebigstr. 20a, 04103 Leipzig, Germany; 6 LIFE Leipzig Research Centre for Civilization Diseases, University of Leipzig, Liebigstr. 20a, 04103 Leipzig, Germany; 7 Institute of Medical Sociology, Medical Faculty, Martin Luther University Halle-Wittenberg, Magdeburger Str. 8, 06112 Halle Saale, Germany

**Keywords:** School performance, Reference group effects, Health complaints, BFLPE, Multilevel analysis, HBSC

## Abstract

**Background:**

Features of schools and classes are closely related to students´ health and wellbeing. However, class composition (e.g. in terms of school performance) has rarely been examined in relation to students´ health and wellbeing. This study focuses on the so called Big-Fish-Little-Pond-Effect (BFLPE), by investigating whether the level of high-performing students in classroom is negatively associated with psychosomatic complaints of students who perceive themselves as poor performers.

**Methods:**

Data were derived from the German sample of the WHO-Collaborative “Health Behaviour in School-aged Children (HBSC)” study 2013/2014. The sample included 5226 11-, 13- and 15-year-old students. Individual perceived school performance (PSP) was included (very good/good vs. average/below average PSP) at the individual student-level. At the class-level, school performance in class was generated by aggregating the share (in percentage) of students who report a very good/good PSP to the class-level, indicating the percentage of students with good/very good PSP in classroom. Using multilevel regression models, the association between class-level school performance (in percentage of students with very good/good PSP) and individual psychosomatic complaints were analyzed, stratified by students´ individual PSP.

**Results:**

Students who report average/below average PSP showed higher likelihoods of psychosomatic complaints (Odds Ratio: 1.75; 95% Confidence Interval: 1.52–2.03) compared to counterparts with very good/good PSP. The aggregated class-level PSP was not significantly associated with psychosomatic complaints. However, in line with the BFLPE, results further revealed that students with average/below average PSP, who attend classes with a higher percentage of students who report very good/good PSP, had higher likelihoods of psychosomatic complaints (Odds Ratio: 1.91; 95% Confidence Interval: 1.01–4.01) compared to classmates with very good/good PSP.

**Conclusions:**

This study revealed that class composition in terms of PSP was differentially associated with students´ psychosomatic complaints, depending on their individual PSP. Findings highlight the vulnerability of students with poor PSP placed in classes with a higher percentage of students with good PSP. Results of this study therefore indicate a need for initiatives for low performing students from teachers and school staff in class.

## Background

Schools represent an important social context for adolescents, where students spend a large proportion of their daily time and with a group of classmates with whom they regularly interact [1]. Many studies have argued that students tend to use their peers in class or school as a reference group to form theirs self-views [2]. Those comparisons are often related to school performance and ability levels among students in school [3].

From studies on social comparison among students in classrooms (see review by Dijkstra et al. [3]) it is evident that most young people compare their abilities with those of others who perform better than themselves, mainly resulting in contrast-effects. As a result, comparisons with better-performing others is likely to lower, for instance, cognitive outcomes, such as academic self-concept, whereas comparing with worse-performing others may enhance those cognitive outcomes [4]. However, there is also an assimilation effect, which is positively related to those outcomes as, for instance, a higher perceived school status allows students to reflect in the glory of their successful school; an effect that is well-known as the “basking in reflected-glory-effect (BIRGE)” [5].

**Fig. 1:**
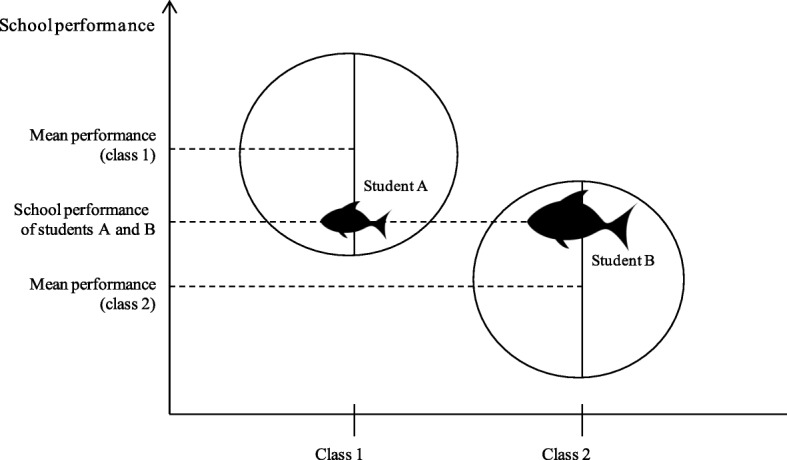
The “Big-Fish-Little-Pond-Effect” (BFLPE) [12]. Modified according to Köller [12]; the size of the fishes represent students’ level of self-concept. Graphics are obtained from http://www.clipartbest.com/clipart-pT5okjabc. The present study used students´ self-reported perceived school performance as data on objective measures of school performance was not available. Thus, the figure only demonstrates the original effect of the Big-Fish-Little-Pond-hypothesis according to previous studies, using test scores or other objective measures of school performance

Comparisons with reference groups have been examined mainly in relation to performance-related composition in classes or schools and its impact on students´ self-concept [6–8], showing a clear contrast-effect, which is well-known as the so-called “Big-Fish-Little-Pond-Effect (BFLPE)”. In its simplest form, the BFLPE predicts that equally performing students have lower academic or general self-concepts when attending schools or classes where the average performance-levels of classmates are high, and higher self-concepts when attending schools or classes where the school- or class-average performance is low [8, 9]. In other words, the same student will have a lower (academic) self-concept (“small fish”, student A) in a group with higher school performance (in a “big pond”, reference group 1) and a higher (academic) self-concept (“big fish”, student B) in a group with lower school performance (in a “small pond”, reference group 2) because this student compares and contrasts his or her own school performance with that of his classmates (Fig. 1). The existence of the BFLPE has been shown in manifold (cross-) national studies that found negative associations between class-level performance and students´ academic or general self-concept among poor-performing students [7, 10, 11].

Marsh [8] reviewed studies showing negative effects of school- or class-average performance levels on a variety of cognitive, behavioral and psychological variables that constitute many of the main outcomes of education, such as educational aspirations, self-concept, school grades or standardized test scores. In general, adolescence is a clearly vulnerable phase when young people are required to fulfill a variety of developmental tasks, particularly in schools and with peer groups. Mismatches between the school environment and the developmental needs and capacities of students [1] may have a negative impact on the mental or psychosomatic health of students [13].

However, with regard to students´ psychosocial health, there is a lack of studies which examined the BFLPE in relation to the association between the percentage of students in schools or classes who report better school performance and students’ individually perceived performance. While young people can generally be considered as healthy in terms of severe chronic diseases, adverse mental health and psychosomatic complaints are already evident in this stage of life [14]. Thus, relating the class-composition in terms of the percentage of students who report better school performance to students’ psychosomatic health is clearly a new facet in the research area of the BFLPE as prior research on the BFLPE mainly focused on different measures of self-concept, but not on indicators of young people’s psychosocial development. Regarding the contrast-effect of the BFLPE-hypothesis, the notion of relative deprivation could be closely linked to young people’s development and psychosomatic health as young people make comparisons with the reference group in class [3, 4, 15]. Feelings of deprivation and incompetence when comparing their perceived school performance with other peers might relate to feelings of lowered self-esteem, inferiority and a worse social standing among other students in class, resulting in psychological problems, such as psychosomatic health complaints.

In line with this argument, the present study examines whether the composition of students who report better school performance in classrooms has a different impact on psychosomatic health complaints of students reporting better or worse school performance. This study focuses on the following research question: Is being a “small fish in a big pond” bad for students´ psychosomatic health complaints?

According to the contrast-effect of social comparison processes in school classes [3], this study tests the hypothesis of the BFLPE in relation to students’ psychosomatic health complaints. More precisely, this study assumes that a student, who perceives his or her school performance as poor (“small fish”), attending a class with a higher number of classmates who report to perceive better school performance (“big pond”), is likely to be exposed as a “small fish” to a “big pond” in terms of comparisons with the “high-performing” reference group of classmates, which might result in poorer psychosomatic health.

## Methods

### Data

Data were obtained from the German arm of the international “Health Behaviour in School-aged Children (HBSC)”-study, a multinational cross-sectional and representative survey conducted in collaboration with the World Health Organization [14]. The aim of the HBSC-study is to examine young people’s health and health behavior and to identify how these outcomes are related to social contexts. Cross-sectional surveys are carried out every four years in a growing number of countries based on an internationally agreed protocol [16, 17]. Starting in 1983 with 4 countries, the latest survey in 2013/2014 included a total of 44 countries in Europe, North America, and Israel. A detailed description of the aims and theoretical framework of the study can be found elsewhere [14]. Students were selected using a clustered sampling design, where the initial sampling unit was the school class. The recommended minimum sample size for each country was 1536 students per age group (i.e. 11-, 13- and 15- year olds), to assure a 95% confidence interval of +/− 3% for prevalence estimates of around 50%. The sample size included a design effect of 1.2 because of the cluster sampling (the design factor of 1.2 was based on analyses of the 1993/1994 and 1997/1998 HBSC-studies). In some of the participating countries, HBSC was exempt from the requirement for ethical approval. In other countries that required approval this was obtained by different institutional review boards. Unfortunately, HBSC survey data is only available as open access files, approximately four years after completion of data from the HBSC Data Management Centre at the University in Bergen, Norway (for further information, please see Website: http://www.uib.no/en/hbscdata). Before that time, data is available upon request. The data were collected by means of standardized questionnaires with around 40 mandatory questions, administered in school classrooms according to standard instructions [14].

The German HBSC-study 2013/2014 is based on a representative sample of federal states. Students were selected using a clustered sampling design [18]. Schools were sampled randomly from a list of public schools, stratified by type of school and administrative district. The raw sample of the German HBSC-study contained 744 randomly selected schools, but only 188 out of 744 selected schools took part in the German HSBC-study 2013/2014, indicating a response rate at the school-level of 24.4%. Thus, 5961 students nested in 557 school classes and 188 schools were sampled in total. The individual response rate was 77.2% for students formally enrolled in the participating schools. As in Germany students are taught in age-homogeneous classes, pupils from grades 5, 7, and 9 were included, representing the age groups of 11-, 13-, and 15- year-olds. Data were collected by means of a standardized questionnaire. Those students were included in the study who had volunteered to participate and whose parents had also signed an informed consent [18, 19]. The study was approved by the federal data protection commissioner of each federal state.

### Sample

The final analyses are based on a total of 5226 students, representing 87.7% of the original sample nested in 172 schools as well as 471 classes (grade 5: *n* = 1439; grade 7: *n* = 1872; grade 9: *n* = 1915). Students, whose information on the indicators used in this study was missing in the database, were excluded from the analyses as complete cases. In total, *n* = 530 students were excluded because information on age (*n* = 51, 0.86%), gender (0%), migration background (*n* = 8, 0.13%), family affluence (*n* = 291, 4.88%), perceived school performance (*n* = 65, 1.09%) or psychosomatic health complaints (*n* = 115, 1.93%) were not available. Classes with a number of less than five students who were allowed to fill out the questionnaire at the survey day, have been excluded (*n* = 205 students, 3.41%) in order to represent a more realistic class average [20]. On average, around 11 students attend a class; the minimum number of students was fixed at five students per class, the maximum number per class was 24 students. At the school-level, 172 schools were included in the analyses, with on average 30 students per school, and 80 students was the maximum number of students per school.

### Indicators

#### Outcome

The HBSC-SCL is a validated measure of psychosomatic health complaints [14, 21]. Students were asked how often in the last 6 months they had experienced the following eight symptoms: headache; stomach ache; backache; feeling low; irritable or bad tempered; feeling nervous; difficulties in getting to sleep, and feeling dizzy. The response options were “almost daily”, “several times per week”, “almost every week”, “about once per month”, “rarely or never.” Each item has been dichotomized (0 = less than several times per week vs. 1 = at least several times per week) and then summed up in a sum index, indicating the number of at least weekly psychosomatic health complaints was calculated from the eight items (range: 0–8 health complaints at least weekly). According to the common HBSC procedures in national and international studies, this index was then dichotomized (1 = 2+ psychosomatic health complaints at least weekly vs. 0 = < 2 complaints) [14].

#### Individual school performance

Students´ individual school performance was measured by their perceived school performance (PSP), because the HBSC^−^study is a cross-national study, conducted in countries with different school and grading systems [22]. Instead of asking students about their school grades, the HBSC students’ questionnaire used the perceived school performance as an indicator that reflects student’s actual school grades [22]. Students were asked *“what, in your opinion, your class teacher(s) do/does think(s) about your school performance compared to your classmates?”* Response options “very good”, “good”, “average” and “below average” [14] were categorized by combining the first and last two options (“very good/good” used as reference category vs. “average/below average”).

#### School performance at the class-level

In order to investigate the BFLPE, individuals´ school performance was aggregated to the class-level as a proportion of students with very good/good school performance in class, ranging from 0 to 100% of students with “high school performance” in class. This aggregated proportion has then been centered around the Grand mean in order to make coefficients of the average-level of PSP in class comparable across the entire sample.

#### Control variables

As psychosomatic health among young people substantially differ among students with different socioeconomic and socio-demographic backgrounds, this study controlled for age (centered on the Grand mean of the study sample) and gender (boys as reference category), migration background, as well as the attended school type and family affluence. Migration background was assessed by asking students about their and parents country of birth as well as language spoken at home [23], creating a categorical variable (no, one-sided and two-sided migration background). Students with one parent born abroad or with other nationality than German have “one-sided” migration background. Students with a “two-sided” migration background are students who are born abroad and have at least one parent who was born abroad, or who have parents who were both migrated to Germany or have another nationality than German. Missing information on students´ or parents´ country of birth was substituted by spoken language at home.

This study further controlled for the attended school type as the German educational system is well known for its “tripartite” structure, indicating a co-existence of different tracks in secondary education with different requirements and learning environments, and its high social segregation among different tracks (i.e. the transition to a secondary track is strongly associated with the social background of students). The most basic type is secondary general school (low educational track, “Hauptschule”), followed by the relatively more advanced intermediate school (medium educational track, “Realschule”), and the most advanced grammar school (high educational track, “Gymnasium”) with a final examination that qualifies for university entrance. Besides the three tracks, a fourth school type exists which does not fit completely into the hierarchically structured system. This comprehensive school (mixed track) unites students of all levels of school performances under one roof and offers options for all three “tracks” above. Further, also schools for students with special educational needs were considered in the analyses. These five school types were dichotomized into “high track schools” (as reference category) versus “low track schools” (low, medium and mixed track as well as schools for students with special educational needs).

Socioeconomic background was measured by the family affluence, using the family affluence scale (FAS) which is a validated measure based on four different aspects of the household’s material conditions [16, 24, 25]: Does your family own a car? (0, 1, 2 or more); how many times did you travel away on holiday with your family during the past 12 months? (0, 1, 2, 3 or more); do you have your own bedroom for yourself? (no = 0, yes = 1); and how many computers does your family own? (0, 1, 2, 3 or more). A composite FAS score was calculated by summing the responses to these four items ranging from 0 to 9. The FAS scores were subsequently recoded in three groups: high (7–9 points), middle (4–6 points) and low (0–3). Internal consistency between items of the family affluence scale was moderate (Cronbachs alpha: 0.50), ranging from a minimum values of 1 to a maximum value of 7 and with a mean value of 4.90 (SD: 2.1). Family affluence has several benefits such as the low percentage of missing responses from young people and documented cross-national comparability [16, 17].

### Statistical analyses

The study conducted three-level multilevel analysis that allows the modeling of hierarchical or nested data structures. The level 1-units in the sample are individual students; the level 2-units are classes and level 3-units are schools. Analyses were conducted by using three-level random intercept models, allowing the intercept of psychosomatic health complaints to randomly vary among levels of individuals, classes and schools. The extent to which the intercept of the outcome differed among schools and classrooms was examined by conducting logistic random intercept models [26]. Individual- and class-level variables have been included in the models that might explain this variation in psychosomatic health complaints, by a stepwise approach.

The individual- and class-level variables were included in the models using a stepwise approach. First, an empty model (Model 1) tested the (pseudo-) Intraclass-Correlation-Coefficient (ICC) as this study applied logistic multilevel models [20], which represents the proportion of variance on latent school and classroom effects by indicating the variance in the outcome attributed to differences between schools and classes [20, 26]. Model 2 considered only individual indicators. In a next step, the proportion of students with very good/good PSP at the class-level was included (Model 3). Finally, model 4 tests the BFLPE, by introducing a cross-level interaction term between the proportion of students with very good/good PSP at the class-level and students´ individual PSP in order to examine their differential impact on psychosomatic health complaints. Models 2–4 controlled for socio-demographic and socioeconomic variables as well as the attended school type. The statistical analyses were conducted using the software Stata 13.1 (StataCorp LP, College Station, TX).

## Results

### Descriptive results

Table 1 shows the bivariate results of psychosomatic health complaints, stratified by individual PSP and proportion of students with very good/good PSP at the class-level. In total, around 24% of students reported psychosomatic health complaints. Prevalence rates in health complaints varied significantly by students’ individual PSP, showing that 29% of students with poor PSP revealed health complaints compared to 19% of students with better PSP (*p* < 0.001; χ^2^ = 22.60, df = 1).

**Table 1:** Descriptive results, HBSC Germany 2013/2014 (n = 5226)

	2+ psychosomatic health complaints	< 1 psychosomatic health complaints	Total
	% (n)	% (n)	% (n)
Gender	*p* < 0.001 (χ^2^ = 146.23, df = 1)		
Boys	17.04 (452)	82.96 (2200)	50.75 (2652)
Girls	31.35 (807)	68.65 (1767)	49.25 (2574)
Age (m = 13.56, SD = 1.64)	*p* < 0.001 (χ^2^ = 62.60, df = 1)		
< Mean age (10.50–13.56 years)	19.62 (536)	80.38 (2196)	52.28 (2732)
> Mean age (13.57–16.33 years)	28.99 (723)	71.01 (1771)	47.72 (2494)
Migration background	*p* < 0.001 (χ^2^ = 32.02, df = 2)		
No migration background	22.05 (839)	77.95 (2966)	72.81 (3805)
One-sided	30.09 (161)	69.91 (374)	10.24 (535)
Two-sided	29.23 (259)	70.77 (627)	16.95 (886)
Family affluence	*p* = 0.016 (χ^2^ = 8.26, df = 2)		
High	22.87 (496)	77.13 (1673)	41.50 (2169)
Medium	23.95 (551)	76.05 (1750)	44.03 (2301)
Low	28.04 (212)	71.96 (544)	14.47 (756)
School type	*p* = 0.001 (χ^2^ = 10.35, df = 1)		
High track (“Gymnasium”)	21.56 (408)	78.44 (1484)	36.20 (1892)
Other school types	25.52 (851)	74.48 (75.91)	63.80 (3.334)
Perceived school performance (PSP)	*p* < 0.001 (χ^2^ = 75.91, df = 1)		
Very good/good	18.95 (497)	81.05 (2125)	50.17 (2622)
Average/below average	29.26 (762)	70.74 (1842)	49.83 (2604)
Proportion of students reporting very good/good PSP in class	*p* < 0.001 (χ^2^ = 22.60, df = 1)		
> 50% of students with very good/good PSP	21.04 (504)	78.96 (1892)	45.85 (2396)
< 51% of students with very good/good PSP	26.68 (755)	73.32 (2075)	54.15 (2830)
Total	24.09 (1259)	75.91 (3967)	100 (5226)

*PSP* Perceived School Performance

**Fig. 2:**
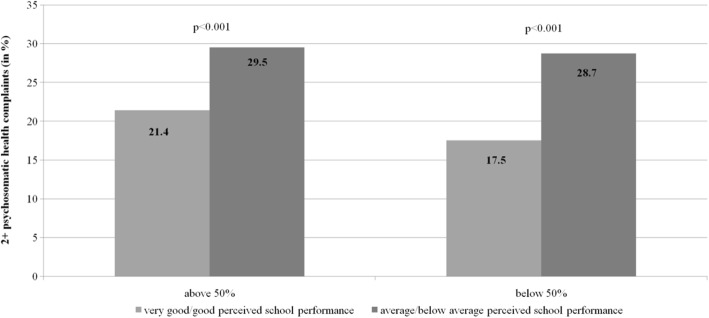
Prevalence of health complaints between classes (> 50% and < 50% of students with very good/good PSP)

Further, students placed in classes with more than 50% of students reporting very good/good PSP showed lower prevalence rates in psychosomatic health complaints (21%) in contrast to students attending classes with a proportion of below 50% of students reporting very good/good PSP (26.7%) (Table 1). Prevalence rates of psychosomatic health complaints for students with good and poor PSP in classes with more than 50% and less than 50% of students who report very good/good PSP (Fig. 2) have been calculated, respectively, in order to test the BFLPE.

As shown in Fig. 2, students who report poor PSP and who are placed in classes with more than 50% of students who report very good/good PSP revealed a significantly higher prevalence rate in psychosomatic health complaints in comparison to counterparts with better PSP. A similar pattern was found in classes with below 50% of students with very good/good PSP, however, with on average lower prevalence rates in psychosomatic health complaints.

### Multivariate results

Table 2 shows the results from the logistic multilevel models for psychosomatic health complaints. According to the (pseudo-) Intraclass-Correlation-Coefficients between schools (ICC = 1.1%) and between classes (ICC = 5.9%) in the empty model (Model 1), the outcome measure does vary among classes and to a lesser amount between schools. Model 2 included individual background characteristics: Girls and older students had significantly higher likelihoods of psychosomatic health complaints compared to their male or younger counterparts, whereas family affluence was not significantly related to the outcome. Further, students with migration background, attending lower track schools and with poor PSP revealed higher odds ratios of reporting psychosomatic health complaints compared to their reference groups. These associations remained stable when the proportion of students with very good/good PSP in classroom was included into models 3 and 4. However, the proportion of students with better PSP in class (Model 3) was not significantly related to psychosomatic health complaints, indicating that the composition in class in terms of the percentage of students with good PSP does not relate to students´ overall psychosomatic health complaints.

**Table 2:** Logistic multilevel results for health complaints

	Model 1: Empty model	Model 2: Individual variables	Model 3: Model 2 + school performance at class-level	Model 4: Cross-level interaction
	OR (95% CI)	OR (95% CI)	OR (95% CI)	OR (95% CI)
Individual variables				
Intercept	0.29*** (0.27–0.33)	0.11*** (0.09–0.14)	0.11*** (0.09–0.14)	0.11*** (0.09–0.14)
Gender (Ref.: Boys)				
Girls		2.39*** (2.08–2.75)	2.39*** (2.08–2.75)	2.39*** (2.08–2.75)
Age (centered)		1.16*** (1.11–1.22)	1.17*** (1.11–1.23)	1.16** (1.10–1.22)
Individual PSP (Ref.: very good/good) average/below average		1.74*** (1.52–2.00)	1.75*** (1.51–2.03)	1.75*** (1.52–2.03)
Family affluence (Ref.: high)				
medium		0.96 (0.83–1.11)	0.96 (0.83–1.11)	0.96 (0.83–1.11)
low		1.09 (0.88–1.34)	1.09 (0.88–1.34)	1.09 (0.88–1.34)
Migration background (Ref.: no)				
One-sided		1.44*** (1.16–1.78)	1.44*** (1.16–1.78)	1.44*** (1.16–1.79)
Two-sided		1.30*** (1.09–1.56)	1.30*** (1.09–1.56)	1.31*** (1.09–1.57)
Class-level variable				
Proportion of students with very good/good school PSP in class (centered)			1.04 (0.67–1.59)	1.31 (0.41–1.32)
Cross-level interaction				
Proportion of students with very good/good PSP in class (centered) x individual school performance (Ref.: very good/good)				
x average/below average individual PSP				1.91* (1.01-4.01)
School-level variable				
School type (Ref.: high track)		1.24**	1.24**	1.25**
Other school types		(1.04–1.48)	(1.04–1.48)	(1.05–1.49)
Variance in intercept (between classes)	0.212***	0.110***	0.110***	0.108***
Variance in intercept (between schools)	0.037	0.050	0.050	0.049
ICC (Class)	0.059 = 5.9%	0.032 = 3.2%	0.032 = 3.2%	0.031 = 3.1%
ICC (school)	0.011 = 1.1%	0.015 = 1.5%	0.015 = 1.5%	0.014 = 1.4%

The class-level variable “proportion of students with very good/good school performance in class” (in %) has been centered around the Grand mean. PSP=Perceived School Performance

*p < 0.050

**p < 0.010

***p < 0.001

With regard to the BFLPE, Model 4 includes cross-level interaction terms between the proportion of students with good PSP and students´ individual PSP in order to examine whether the performance-related environment in class is differentially related to young people’s likelihoods of psychosomatic health complaints.

**Fig. 3:**
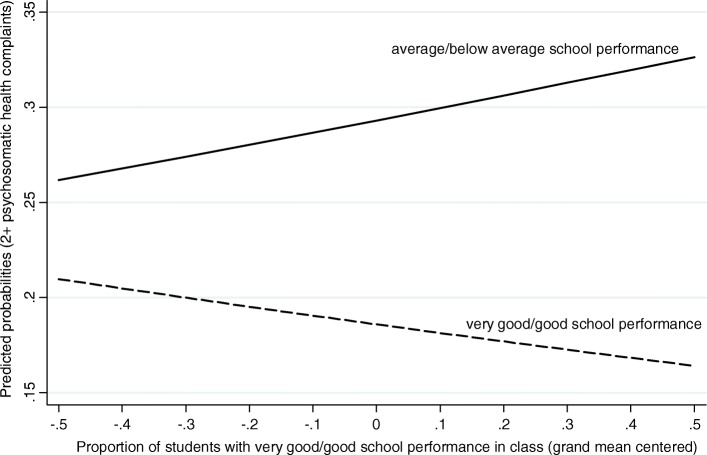
Predicted probabilities of health complaints in relation to PSP at the class-level (HBSC 2013/2014, *n* = 5226)

In line with the BFLPE-hypothesis, the results show that students perceiving their school performance as poor revealed higher likelihoods of reporting psychosomatic health complaints, when they attend classrooms with a higher proportion of students perceiving their school performance as good (Fig. 3). In contrast, the findings of this study revealed higher probabilities of psychosomatic health complaints for students with good PSP, when they are placed in classrooms with a lower proportion of classmates with high PSP.

## Discussion

### Synthesis of the findings

Our study is among the first analyzing the impact of the so-called BFLPE on students´ psychosomatic health complaints. The BFLPE has originally been tested in studies on students´ general or academic self-concept, hypothesizing that students primarily compare their own school performance with that of their school- or classmates and use this social comparison information of their reference groups as the basis of their own academic self-concept [6, 27]. In line with previous research, this study extended the prior state-of-the-art, by revealing a contrast-effect which supports the BFLPE-hypothesis. The results showed a negative impact on psychosomatic health complaints for students with poor PSP, when they are placed in classes with a higher percentage of classmates reporting good PSP.

First of all, this study showed that students who reported poor PSP revealed higher likelihoods of psychosomatic health complaints. This finding is very much in line with other studies, focusing on young people’s academic achievement and health outcomes. Those studies revealed significant links between low academic performance at school and low levels in health-related outcomes, such as self-rated health and well-being [14, 28, 29]. In general, school performance is closely related to the educational background and competence levels of young people [30]. Many studies highlighted that, for instance, high levels of education increase the sense of personal control, the belief that one can master, control, or effectively alter the environment [31], while poorly educated (young) people may not possess the resources necessary to achieve their goals, being related to a sense of powerlessness and learned helplessness [32]. In this context, students who report poor PSP are likely to fare worse at school in terms of performance and school grades [22]. The social, cultural and intellectual resources provided by education also contribute to health-promoting choices [30]. It is therefore possible that in a society where education is highly valued and strongly dependent on students socioeconomic background characteristics, such as Germany, the perception of low school performance in school might be a stressor for a young person as poor school performance may deteriorate self-confidence and lead to anxiety, which is measurable by health indicators, such as psychosomatic health complaints [33–35].

However, the relationship between school performance and psychosomatic health complaints could also be reversed. Therefore, it could also be the case that poor health would be associated with poorer school performance or that poor health could be connected with an underestimation of one’s own school performance. Unfortunately, these associations cannot be completely unraveled as this study used cross-sectional data. Prior studies recently started to unravel this causal mechanism and highlighted that children who are unhealthy or report poor health are at higher risk for school problems, have a higher probability of school failure, grade retention, and dropout than students who are free from medical problems or report good health (see review by [36]. However, a review on the relationship between student health and academic success concluded that this association is rather complex [36]. In order to clarify the causal chain between school performance and students psychosomatic health, future studies using longitudinal data are warranted.

With regard to our main research question about whether being a “small fish in a big pond” is bad for students´ psychosomatic health complaints, the findings are in line with this hypothesis of the BFLPE- or contrast-effect. However, as prior studies have not focused on the BFLPE in relation to students´ psychosomatic health, it is only possible to compare findings of this study with results from previous research on self-concept. This study revealed that there was no overall significant association between the percentage of students with very good/good PSP and psychosomatic health complaints. Other studies, for instance, on academic self-concept also showed that the contrast-effect did not apply for all students in the same way [15]. In line with their findings, our study revealed that students who report poor PSP were negatively affected by the proportion of classmates reporting very good/good PSP in class. This pattern is very reasonable for students who report poor PSP as they are likely to belong to a minority and most likely stigmatized group in classes where the majority of classmates perform well at school. Previous studies have shown that having a stigmatized position in a context where, for instance, different status groups interact regularly is likely to negatively impact on wellbeing or psychosomatic health [37]. A similar mechanism can be applied for students with poor PSP being placed in classes with high-performing counterparts as they compare themselves with the reference group [27] and struggle with maintaining their school performance as well as possible. Regarding the stage-environment-fit approach [1], this stigmatized position in the classroom is likely to challenge students’ psychological needs for competence and relatedness.

Although we cannot proof this explanatory mechanism with our data, it may further be likely that students reporting poor PSP may discern pressure, e.g. from teachers or from better performing classmates in order to keep up with them. Overall, this finding highlights the importance of the learning environment, which is a commonly reported source of distress among young people [34, 38–40], particularly for those who are not performing very well at school. In line with other studies, feelings of pressure at school are associated with more frequent somatic (headache, abdominal pain, backache and dizziness) and psychological health complaints (feeling sad, tense and nervous) [34, 41], with lower self-reported health, life satisfaction or well-being [14, 34, 35, 39, 42, 43].

In contrast to students reporting average/below average PSP, the results further showed that students revealing very good/good PSP were not related to the negative contrast-effect when they were placed in classes with a high proportion of students who report very good/good PSP. From a theoretical point of view, this finding is quite reasonable. Students who report good PSP may have less reason to be afraid of other well performing classmates as they still fare quite well in a socially competitive arena [8, 15]. Being surrounded by other classmates who report good PSP might not negatively affect those students in terms of psychosomatic health complaints. However, it is further likely that students with good PSP, who are surrounded by classmates who are also good at school, may be more likely to be under pressure to maintain their school performance at a good level, which could negatively affect their psychosomatic health. Further studies are therefore required in order to retest and validate the findings.

### Strengths and limitations

A strength of this study is that it used a large representative sample of secondary school students in Germany, with students nested within classes and classes nested within schools. This data structure, which is quite uncommon in these types of studies, makes it possible to carry out analyses at three different levels of the school structure and to take compositional measures at the class-level into account.

However, both predictor and outcome variables rely on self-reported data, which raises the issue of negative affectivity [44]. In contrast, previous studies on the BFLPE have mostly used standardized achievement tests in order to measure students´ school performance [2], while some other studies also used self-reports of school performance or perceived standing of students in specific subjects [15], showing in either ways, a negative frame-of-reference-effect for students’ academic or general self-concept. It is further likely that many factors, such as idiosyncratic classroom norms for performance and investment in schoolwork or classroom climate, may be related to students’ perceptions of their performance.

Unfortunately, the HBSC-study only surveys the perceived school performance (PSP) of students, which does not completely reflect the actual school performance, and thus, is likely to be influenced by non-academic factors such as the teacher’s relationship with the student (e.g. feeling more accepted). However, it is surprising that the measure of PSP, as it is used in the international and German HBSC-study, highly correlates with students actual school grades (see validation study by [22]). This validation study concluded that the PSP item seems to be a valid and useful question that can distinguish groups of respondents that receive good grades at school from those that do not. Therefore, this study used the percentage of students perceiving their school performance as very good/good as a proxy of the mean school performance in classes, although there are limitations by using this indicator of PSP.

Nevertheless, the HBSC-study is a unique data base, because it not only provides information from students about self-rated health outcomes, but also provides hierarchically structured data in order to test multilevel proposals by considering the class- or school-level as central environments of young people’s health and overall development. In comparison to other surveys on young people’s health in Germany, the HBSC-study is the only database which surveys students in school classes and schools, and, thus, allowing to relate those learning environments to students’ health and wellbeing. In order to test the BFPLE- or contrast-effect for students´ psychosomatic health, this study had to rely on this data source and the measure of PSP because the HBSC-study did not survey grade point averages from students. However, relating the class-composition in terms of the percentage of students who perceive better school performance to students’ psychosomatic health is a clearly new facet in the research area of the BFLPE as prior research on the BFLPE mainly focused on different measures of self-concept, but not on indicators of young people’s psychosocial development. Other measures of school performance, such as grade point averages or particularly standardized competence tests would be much more desirable and reliable in order to examine the BFLPE in relation to students´ psychosomatic health, but are not available in the HBSC-survey. Lastly, even studies measuring school grades, most of those studies rely on self-reported measures from students instead of surveying competence tests.

Regarding the self-reported measures of family affluence, this study used the so-called Family Affluence Scale (FAS), an index measure of young people’s material family wealth, which has been validated several times [16] and shows strong correlations with parental income. Further, the average FAS of students in a country correspond with objective measures of wealth in the country, such as GNI [24, 25]. Andersen et al. [45] described high agreement between parents´ and 11 yearold students´ response on the FAS items. Another study from Ireland showed that FAS revealed a moderate internal reliability and FAS scores were significantly associated with reported parental occupation [46]. Lastly, the family affluence scale has the benefit that young people can easily respond to the items in relation to the material situation at home, and thus, a low percentage of missing responses from young people is more likely in contrast to parental socioeconomic measures, such as parental income or educational level [16, 17]. Further, this study used a cross-sectional design, not allowing for assessment of causal inferences.

In contrast to many other studies on the BFLPE, control variables have been used when testing the interaction term between class-level school performance and students´ individual school performance. However, this study did not consider possible mediator variables, such as students’ perception of teaching characteristics or school climate, which might negatively be related to the strength of the interaction term [47, 48]. Although the majority of studies on the Big-Fish-Little-Pond-Effect (BFLPE), carried out by Herbert Marsh and colleagues, did not test mediating or moderating effects, it is further likely that the association between students´ individual school performance and the overall school performance in school classes is mediated or moderated by other school-related features, such as school connectedness. Here, prior studies revealed that students who report higher school connectedness, showed better school performance, also in a longitudinal perspective [49]. In sum, further research is warranted on the conditions under which individual PSP buffers the impact of class-level school performance, particularly for students who perceive themselves as performing poorly at school, and by using a longitudinal design.

Another limitation is related to the low response rate at the school-level and the level of eligible participants in our sample. Therefore, further caution is required when generalizing study results.

The results of this study further showed that almost all of the variability in psychosomatic health complaints between schools and classes was explained by differences between individuals. This confirms previous multilevel research on subjective health and wellbeing among students [50–53]. Other studies also showed that the extent to which variation in student outcomes was conditioned by differences between schools or classes, and varied according to the outcome under study [53, 54]. For instance, studies on students’ health behaviors reported an ICC of 7–12% [54], whereas studies on students’ wellbeing generally reported much lower ICCs between schools and classrooms [50, 51, 55]. Further, variance in psychosomatic health complaints was greater between classes compared to the school-level. In Germany, school-aged children in lower secondary school (grades 5, 7 and 9) are generally grouped with the same classmates for the majority of school subjects during school day. Ability- or subject-related grouping is mainly practiced in higher secondary education. Thus, observing a higher ICC between classes compared to schools indicates that there is, however, more variation in psychosomatic health complaints between classes than schools. This could be due to different learning environments in classes in terms of the teaching and learning climate or teacher-student-relationship.

## Conclusions

This study focused for the first time on the so-called BFLPE, by examining the differential association between the proportion of students with good PSP in classrooms (“big ponds”) and psychosomatic health complaints of students who perceive their school performance as poor (“small fish”). The main aim of this study was to examine whether being a “small fish in a big pond” is detrimental to students´ psychosomatic health. According to the results of this study, students with poor PSP (“small fishes”) are more likely to report health complaints if they are placed in classes with a high proportion of students with good PSP (“big ponds”). Thus, the findings suggest a certain vulnerability of students perceiving themselves as poor performers compared to classmates and particularly when they were placed in classrooms where they were surrounded by a higher percentage of classmates who report better PSP. This indicates a need for school initiatives, undertaken for example by school psychologists and teachers, which emphasize not only students’ academic performances, but also their health and wellbeing. For instance, among others (see Table 3), schools and teachers are requested, to facilitate within-class grouping, aiming not only at grouping students by similar performance levels, rather than mixing students with different performance-levels in heterogeneous learning groups within classes.

**Table 3:** Practical recommendations for researchers, teachers and school administrators

Researchers	• To investigate the association between possible mediator or moderator indicators (e.g. school connectedness, school burnout, etc.) • To include more objective measures of school performance and educational attainment in Health Surveys with children and adolescents. • Longitudinal studies: To examine trajectories of health and wellbeing throughout the school career in relation to students´ individual school performance and with regard to the class environment where students learn.
Teachers	• Being aware of the role that class composition plays for students´ wellbeing. • Put emphasis on students´ individual strengths rather than on their weaknesses. • Create a positive learning environment in class and working groups. • Special support for school-aged children who belong to the low-performers in class (e.g. by special lessons or support in school subjects where children are in need) • Organize learning groups in class with heterogeneous group compositions in order to allow for interaction and support between students with different backgrounds and levels of school performance.
School administrators	• To focus not only on students´ educational success in school, but also on students´ overall wellbeing. • The intake of students with different levels of educational backgrounds requires the awareness of school principals and teachers to organize school work and classes in heterogeneous and mixed learning groups.

Thereby, students with poor PSP could notice that there are other classmates in their group who perceive themselves as poor performers, while students with better PSP could offer help and support to their peers. Further, teachers and school psychologists can also attempt to strengthen students´ self-confidence and academic competences by focusing on their personal and social strengths and resources. It is likely that school performances play an important role in shaping relationships between teachers and students as well as relationships between students. Thus, teachers are encouraged to establish positive learning environments which are characterized by a culture of acceptance and where students are perceived as individuals with their variety of social and emotional personality traits, rather than a learning culture that is shaped by a strong focus only on students´ academic performance and development. School initiatives by teachers and psychologists could further focus on students´ psychosomatic problems and ill-health, by not only emphasizing risk factors, such as school requirements, but also students´ individual factors, such as academic self-concept.

## Abbreviations

**BFLPE**: Big-Fish-Little-Pond-Effect
**FAS**: Family Affluence Scale
**HBSC**: Health Behaviour in School-aged Children
**ICC**: Intraclass-Correlation-Coefficient
**PSP**: Perceived school performance
